# Class I and Class II Histone Deacetylases Are Potential Therapeutic Targets for Treating Pancreatic Cancer

**DOI:** 10.1371/journal.pone.0052095

**Published:** 2012-12-14

**Authors:** Guan Wang, Jing He, Jianyun Zhao, Wenting Yun, Chengzhi Xie, Jeffrey W. Taub, Asfar Azmi, Ramzi M. Mohammad, Yan Dong, Wei Kong, Yingjie Guo, Yubin Ge

**Affiliations:** 1 The State Engineering Laboratory of AIDS Vaccine, College of Life Science, Jilin University, Changchun, China; 2 Division of Pediatric Hematology/Oncology, Children's Hospital of Michigan, Detroit, Michigan, United States of America; 3 Department of Pediatrics, Wayne State University School of Medicine, Detroit, Michigan, United States of America; 4 Molecular Therapeutics Program, Barbara Ann Karmanos Cancer Institute, Wayne State University School of Medicine, Detroit, Michigan, United States of America; 5 Department of Structural and Cellular Biology, Tulane University School of Medicine, Tulane Cancer Center, New Orleans, Louisiana, United States of America; 6 Key Laboratory for Molecular Enzymology and Engineering of the Ministry of Education, Jilin University, Changchun, China; 7 Department of Oncology, Wayne State University School of Medicine, Detroit, Michigan, United States of America; University of Nebraska Medical Center, United States of America

## Abstract

**Background:**

Pancreatic cancer is a highly malignant disease with an extremely poor prognosis. Histone deacetylase inhibitors (HDACIs) have shown promising antitumor activities against preclinical models of pancreatic cancer, either alone or in combination with chemotherapeutic agents. In this study, we sought to identify clinically relevant histone deacetylases (HDACs) to guide the selection of HDAC inhibitors (HDACIs) tailored to the treatment of pancreatic cancer.

**Methodology:**

HDAC expression in seven pancreatic cancer cell lines and normal human pancreatic ductal epithelial cells was determined by Western blotting. Antitumor interactions between class I- and class II-selective HDACIs were determined by MTT assays and standard isobologram/CompuSyn software analyses. The effects of HDACIs on cell death, apoptosis and cell cycle progression, and histone H4, alpha-tubulin, p21, and γH2AX levels were determined by colony formation assays, flow cytometry analysis, and Western blotting, respectively.

**Results:**

The majority of classes I and II HDACs were detected in the pancreatic cancer cell lines, albeit at variable levels. Treatments with MGCD0103 (a class I-selective HDACI) resulted in dose-dependent growth arrest, cell death/apoptosis, and cell cycle arrest in G2/M phase, accompanied by induction of p21 and DNA double-strand breaks (DSBs). In contrast, MC1568 (a class IIa-selective HDACI) or Tubastatin A (a HDAC6-selective inhibitor) showed minimal effects. When combined simultaneously, MC1568 significantly enhanced MGCD0103-induced growth arrest, cell death/apoptosis, and G2/M cell cycle arrest, while Tubastatin A only synergistically enhanced MGCD0103-induced growth arrest. Although MC1568 or Tubastatin A alone had no obvious effects on DNA DSBs and p21 expression, their combination with MGCD0103 resulted in cooperative induction of p21 in the cells.

**Conclusion:**

Our results suggest that classes I and II HDACs are potential therapeutic targets for treating pancreatic cancer. Accordingly, treating pancreatic cancer with pan-HDACIs may be more beneficial than class- or isoform-selective inhibitors.

## Introduction

Pancreatic cancer is a highly malignant disease with a steadily increasing incidence. Despite being the fourth leading cause of death from cancer in the US, little improvement in prognosis has been made over the past 20 years [1]–[3]. Due to delays in clinical diagnosis, pancreatic cancer is often detected at an advanced stage and the prognosis is extremely poor, with a survival of 4 to 6 months [2]. Gemcitabine (2′, 2′-difluorodeoxycytidine, dFdC) is the standard first-line drug for treating patients with advanced pancreatic cancer [4]. However, with median survival of 5.7 months and 1-year survival rate of 18%, its efficacy remains low [5], [6]. Therefore, pancreatic cancer remains a highly chemoresistant malignancy and urgently needs new therapeutic approaches.

Histone deacetylases (HDACs) play critical roles in the epigenetic regulation of gene expression by catalyzing the removal of acetyl groups, stimulating chromatin condensation and promoting transcriptional repression [7], [8]. HDACs comprise a large group of proteins divided into four classes based on their homologies to yeast HDACs, their subcellular localization and their enzymatic activities [8]–[10]. Class I comprises HDAC1, 2, 3 and 8, which are all homologues of the yeast rpd3 protein. They are ubiquitously expressed and located primarily in the nucleus [8]–[10]. Class II enzymes include HDAC4, 5, 6, 7, 9 and 10, which are homologues of the yeast hda1 protein. These enzymes generally exhibit tissue-specific expression and shuttle between the cytoplasm and nucleus in response to cellular signals [8], [11]. Since HDACs 6 and 10 contain two catalytic sites, these enzymes are sometimes further designated as a separate subclass (Class IIb) from HDACs 4, 5, 7, and 9 (Class IIa) [8], [12]. Class III comprises the seven sirtuins, SIRT1-7, homologues of the yeast SIR2 protein [8], [13]. HDAC11 contains conserved residues that are shared by both class I and class II enzymes and represents a separate class of HDAC (Class IV) [8], [10], [14].

Aberrant epigenetic changes are a hallmark of human cancers [15]. High HDAC1 expression has been found to correlate with advanced stage lung and pancreatic cancer [16]–[18]. Thus, HDACs may represent promising targets for pharmacological intervention of cancer. Numerous small molecule HDACIs have been developed during the past decade [19], [20], which have shown promising antitumor activities against preclinical models of pancreatic cancer, either alone or in combination with chemotherapeutic or targeted agents [16], [21]–[24]. However, the clinically relevant HDAC isoforms in pancreatic cancer have not been entirely determined. Knockout and siRNA knockdown experiments have suggested that class I HDACs are essential for cancer cell proliferation and survival in contrast to class II HDACs 4 and 7[25], [26]. However, inhibition of the class IIb HDAC6 leads to acetylation and disruption of the chaperone function of heat-shock 90 (Hsp90) in leukemia cells [27]. Although some HDACIs are considered to be pan-HDACIs (e.g., LBH-589, PXD-101, and SAHA), a recent study demonstrated that the class IIa enzymes are not targeted by most HDACIs (e.g., FK-228, LBH-589, MGCD0103, MS-275, PXD-101, and SAHA) at pharmacologically relevant concentrations [28]. Thus, although it is increasingly apparent that the class I HDAC enzymes are clinically relevant for cancer [25], [26], this is less established for the class II enzymes especially in the context with class I HDACs.

In this study, we examined the expression of classes I and II HDACs in seven pancreatic cancer cell lines and human pancreatic ductal epithelial cells and determined their therapeutic roles in pancreatic cancer cells by using class-, subclass-, and isoform-selective HDACIs. Our results demonstrate, for the first time, *in vitro* synergistic antitumor interactions between class I and class II HDACIs in pancreatic cancer cells, but not in normal human pancreatic ductal epithelial cells. Although there is a need for follow-up studies in *in vivo* models, our results suggest that both class I and class II HDACs are potential therapeutic targets for treating pancreatic cancer.

## Materials and Methods

### HDACIs

The novel class I-selective HDACI MGCD0103 (Mocetinostat) [29], and the class IIa-selective HDACI MC1568 [30]–[32] were purchased from Selleck Chemicals LLC (Houston, TX). The novel HDAC6-selective inhibitor Tubastatin A [33] was purchased from BioVision Inc. (Mountain View, CA). All the HDACIs were dissolved in DMSO and stored at −80°C, as recommended by the suppliers.

### Cell Culture

The HPAC, MIAPaCa-2, BxPC-3, PANC-1 (derived from primary tumor [34]), AsPC-1, CFPAC-1, and Capan-1 (derived from metastasis [34]) human pancreatic cancer cell lines were purchased from the American Type Culture Collection (ATCC; Manassas, VA). Normal human pancreatic ductal epithelial (HPDE) cells were obtained from M. D. Anderson Cancer Center. The cell lines were cultured in Dulbecco's Modified Eagle Medium (DMEM) or RPMI1640 (Invitrogen, Carlsbad, CA) with 10% heat-inactivated fetal bovine serum (FBS; Hyclone Labs, Logan, UT) plus 100 U/mL penicillin and 100 µg/mL streptomycin in a 37°C humidified atmosphere containing 5% CO_2_/95% air.

### *In Vitro* Cytotoxicity Assays

*In vitro* HDACI cytotoxicities of pancreatic cancer cell lines and the HPDE cells were measured by using MTT (3-[4,5-dimethyl-thiazol-2-yl]-2,5-diphenyltetrazolium-bromide, Sigma-Aldrich, St Louis, MO) reagent, as previously described [35], [36]. Briefly, AsPC-1, BxPC-3, PANC-1 (widely used cell line models for pancreatic cancer research), or HPDE cells were cultured in 100 µl of DMEM/10% FBS in 96-well plates. Cells were incubated at 37°C in the presence of 5 variable concentrations of MGCD0103 (0–4 µM), MC1568 (0–10 µM), or Tubastatin A (0–8 µM). After 96 h, MTT was added to a final concentration of 1 mM. After 4.5 hours, formazan crystals were dissolved by the addition of 100 µl of 10% SDS in 10 mM HCl. Optical densities were measured with a visible microplate reader at 590 nm. IC_50_ values were calculated as drug concentrations necessary to inhibit 50% growth compared to untreated control cells. The data for the cell lines are presented as means ± standard errors from at least 3 independent experiments. The extent and direction of MGCD0103 and MC1568 or Tubastatin A antitumor interactions were evaluated by standard isobologram analysis as described previously [35], [37], [38], and by using the CompuSyn software (ComboSyn, Inc., Paramus, NJ). Briefly, drug interactions were quantified by determining the combination index (CI), where CI<1, CI = 1, and CI>1 indicate synergistic, additive, and antagonistic effects, respectively. The data are presented as means ± standard errors from at least 3 independent experiments.

### Colony Formation Assays

One day prior to HDACI treatments, 300 PANC-1 or 500 BxPC-3 cells were seeded into 100 mm dishes in complete DMEM or RPMI160. The cells were then treated with variable concentrations of MGCD0103 (0–1.0 µM), MC1568 (0–10 µM), Tubastatin A (0–4 µM), MGCD0103 plus MC1568 (0.5 µM +5 µM), or MGCD0103 plus Tubastatin A (0.5 µM +2 µM) for 96 h. The cells were then washed twice with drug-free DMEM or RPMI1640 and cultured in complete DMEM or RPMI1640 for up to 3 weeks. Colonies were visualized by coomassie blue staining and counted. Results are presented as mean percentages ± standard errors relative to untreated control cells from three independent experiments. Extent and direction of antitumor interactions between MGCD0103 and MC1568 or Tubastatin A were determined by using the CompuSyn software (ComboSyn, Inc.).

### shRNA Knockdown of HDACs in PANC-1 cells

HDAC4 and HDAC6 shRNA lentivirus clones were purchased from the RNAi Consortium (Sigma-Aldrich) and used to infect PANC-1 cells. After selection with puromycin, a pool of infected cells was expanded and tested for HDAC4 or HDAC6 expression by Western blotting (designated HDAC4- or HDAC6-shRNA cells). A pool of cells from the negative control transduction was used as the negative control (designated NTC-shRNA cells).

### Western Blot Analysis

Soluble proteins were extracted from HPAC, MIAPaCa-2, BxPC-3, PANC-1, AsPC-1, CFPAC-1, Capan-1, or HPDE cells, untreated or treated with HDACIs for 96 h, and subjected to SDS-polyacrylamide gel electrophoresis. Separated proteins were electrophoretically transferred to polyvinylidene difluoride (PVDF) membranes (Thermo Fisher Inc., Rockford, IL) and immunoblotted with anti-HDAC1 (#2062), -HDAC2 (#2540), -HDAC3 (#2632), -HDAC4 (#2072), -HDAC5 (#2082), -HDAC7 (#2882), -p21 (#2947S), -γH2AX (#2577S), (Cell Signaling Technology, Beverly, MA), -HDAC6 (sc-11420, Santa Cruz Biotechnology, Santa Cruz, CA), -HDAC8 (H6412), -HDAC10 (H3412), -acetyl (ac)-tubulin (T7451) (Sigma, Saint Louis, MO), -HDAC9 (SH030228P, ABGENT, San Diego, CA), -ac-histone H4, -histone H4, or -beta-actin antibody (Upstate Biotechnology, Lake Placid, NY), as described previously [39], [40]. Immunoreactive proteins were detected with Lumi-Light Western blotting substrate (Roche Diagnostics, Indianapolis, IN), as described by the manufacturer.

### Assessment of Baseline and HDACI-Induced Apoptosis

BxPC-3 or PANC-1 cells were treated with MGCD0103 (0.5 µM), MC1568 (5 µM), Tubastatin A (2 µM), MGCD0103 plus MC1568 (0.5 µM +5 µM), or MGCD0103 plus Tubastatin A (0.5 µM +2 µM) for 96 h. The cells were then harvested and vigorously pipetted, and samples were taken to determine baseline and HDACI-induced apoptosis using the Apoptosis Annexin V–FITC/Propidium Iodide (PI) kit (Beckman Coulter; Brea, CA) and a FACS Calibur flow cytometer (Becton Dickinson, San Jose, CA), as described previously [35], [36], [41]. Apoptotic events were recorded as a combination of Annexin V^+^/PI^−^ (early apoptotic) and Annexin V^+^/PI^+^ (late apoptotic/dead) events. The experiment was repeated three times, and results are presented as mean percentages ± standard errors of Annexin V^+^ cells of triplicates from one representative experiment.

### Effects of HDACIs on Cell Cycle Progression in Pancreatic Cancer Cells

BxPC-3 or PANC-1 cells treated with MGCD0103 (0.5 µM), MC1568 (5 µM), Tubastatin A (2 µM), MGCD0103 plus MC1568 (0.5 µM +5 µM), or MGCD0103 plus Tubastatin A (0.5 µM +2 µM) for 96 h were harvested and fixed with ice-cold 70% (v/v) ethanol for 24 hours. After centrifugation at 200×g for 5 minutes, the cell pellets were washed with PBS (pH 7.4) and resuspended in PBS containing PI (50 μg/mL), Triton X-100 (0.1%, v/v), and DNase-free RNase (1 μg/mL). DNA contents were determined by flow cytometry (FACS Calibur). Cell cycle analysis was performed with the ModFit *LT* ^TM^3.0 DNA analysis software (Becton Dickinson).

### Statistical Analysis

Differences in MGCD0103 IC_50_s between MC1568 or Tubastatin A treated and untreated BxPC-3 or PANC-1 cells and differences in cell death/apoptosis between MGCD0103 and MC1568 or Tubastatin A treated (individually or combined) and untreated cells were compared using the paired t-test. Statistical analyses were performed with GraphPad Prism 4.0.

## Results

### HDAC Expression and HDACI Sensitivities in Pancreatic Cancer Cell Lines and the HPDE Cells

**Figure 1:**
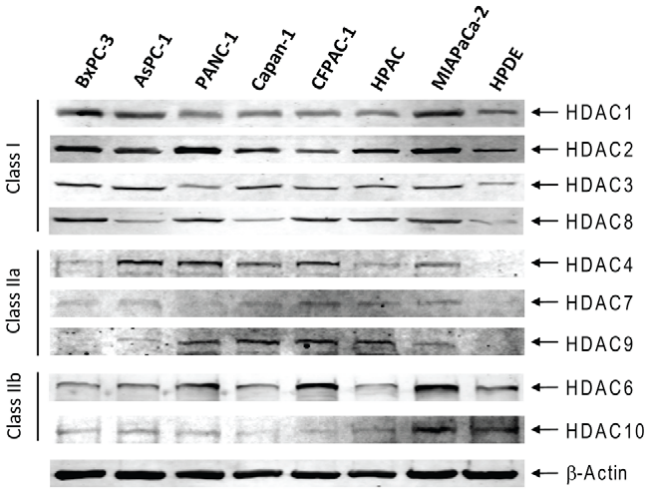
HDAC expression in pancreatic cancer cell lines and the HPDE cells. Protein extracts from log phase AsPC-1, BxPC-3, PANC-1, HPAC, MIAPaCa-2, CFPAC-1, Capan-1, and the HPDE cells were subjected to Western blots probed by anti-HDAC or -β-actin antibody, as described in the Materials and Methods. The class I HDACs (1, 2, 3, and 8) were detected in all the cell lines though the levels were variable. In general, the levels of the class I HDACs in the HPDE cells were relatively lower compared to the majority of the pancreatic cancer cell lines. Interestingly, the majority of class IIa HDACs (except for HDAC5) were detected in almost all the pancreatic cancer cell lines but not in the HPDE cells. In contrast, HDACs 6 and 10 were detected in all the cell lines and their levels in the HPDE cells were comparable (if not higher) to those in the cancer cell lines.

**Figure 2:**
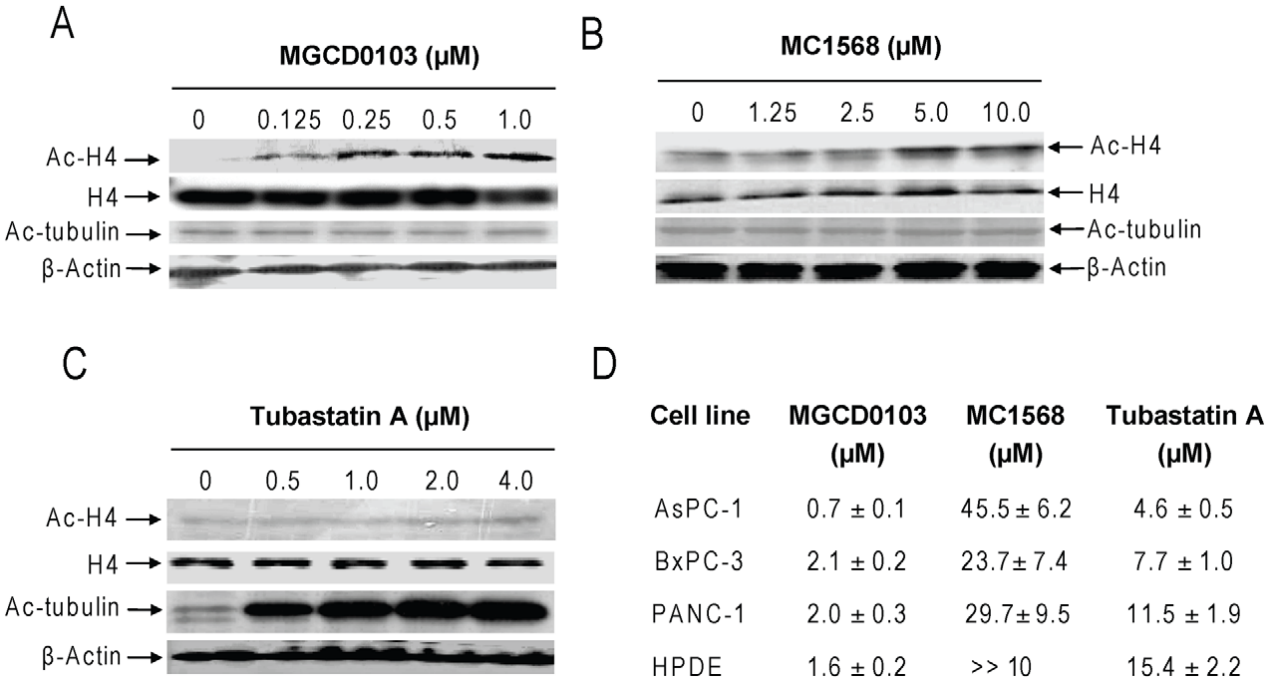
HDACI sensitivities in pancreatic cancer cell lines and the HPDE cells. **Panels A–C:** PANC-1 cells were harvested and lysed after incubation with a range of concentrations of MGCD0103 (0–1.0 µM), MC1568 (0–10 µM), or Tubastatin A (0–4 µM) for 96 h. Soluble proteins were analyzed on Western blots probed by anti-acetylated (ac)-H4, -H4, -ac-tubulin, or –β-actin antibody. **Panel D:** AsPC-1, BxPC-3, PANC-1, or the HPDE cells were cultured at 37°C for 96 h in complete medium in 96-well plates, with a range of concentrations of MGCD0103, MC1568, or Tubastatin A, and cell viabilities were determined using the MTT reagent and a visible light microplate reader. The IC_50_ values were calculated as the concentrations of drug necessary to inhibit 50% growth compared to control cells cultured in the absence of drug. The data are presented as mean values ± standard errors from at least 3 independent experiments. MG, MGCD0103; MC, MC1568; TA, Tubastatin A. The same abbreviations were used throughout the study unless otherwise stated.

The class III HDACs (SIRTs 1–7) are not targeted by traditional HDACIs [20] and were not included in this study. Expression of classes I and II HDACs was determined by Western blotting in HPAC, MIAPaCa-2, BxPC-3, PANC-1, AsPC-1, CFPAC-1, Capan-1, and the normal HPDE cell line. The class I HDACs (1, 2, 3, and 8) were detected in all the cell lines though the levels were variable. In general, the levels of the class I HDACs in the HPDE cells were relatively lower compared to the majority of the pancreatic cancer cell lines. Interestingly, the majority of class IIa HDACs (except for HDAC5) were detected in almost all the pancreatic cancer cell lines but not in the HPDE cells. In contrast, HDACs 6 and 10 were detected in all the cell lines and their levels in the HPDE cells were comparable (if not higher) to those in the cancer cell lines (Figure 1). To determine the roles of these HDACs in pancreatic cancer cell growth and survival, we used three different HDACIs, MGCD0103 (Mocetinostat, a class I-selective HDACI) [29], MC1568 (a class IIa-selective HDACI) [30]–[32], and Tubastatin A (a novel HDAC6-specific inhibitor) [33]. Treatments of PANC-1 cells with variable concentrations of MGCD0103 (0–1.0 µM) resulted in a dose-dependent hyperacetylation of histone H4, while having no effects on alpha-tubulin (a HDAC6 substrate) acetylation or total H4 levels (Figure 2A). Treatments with MC1568 also resulted in acetylation of histone H4 (obvious at 5 and 10 µM), however, to a much lesser extent compared to MGCD0103, and the levels of acetylated histone H4 plateaued at 5 and 10 µM. Further, these treatments had no impact on alpha-tubulin acetylation (Figure 2B). In contrast to the other two HDACIs, Tubastatin A treatments (0–4 µM) resulted in dose-dependent hyperacetylation of alpha-tubulin, which plateaued at 2 and 4 µM. However, these treatments had no effect on the acetylation of histone H4 (Figure 2C). These results validated the HDACI properties of these agents and partially supported their substrate specificities.

Treatments of PANC-1 cells with variable concentrations of MGCD0103 (0–4 µM) resulted in dose-dependent growth arrest, as determined by MTT assays (data not shown), with an IC_50_ of 2.0 µM (Figure 2D). In contrast, treatments of the cells with MC1568 (0–10 µM) or Tubastatin A (0–8 µM) resulted in limited inhibition of cell growth (especially at lower doses, data not shown) with an ***estimated*** IC_50_ of 29.7 µM and 11.5 µM, respectively (Figure 2D). Similar results were obtained with AsPC-1 and BxPC-3 cells (Figure 2D). Surprisingly, the HPDE cells responded to MGCD0103 as well as the pancreatic cancer cell lines (Figure 2D). Although treatments with Tubastatin A (0–4 µM) also resulted in limited inhibition of cell growth of the HPDE cells (with an estimated IC_50_ of 11.5 µM), treatments with MC1568 (0–10 µM) showed no effects at all (data not shown and Figure 2D).

These findings suggest that class I HDACs play critical roles in pancreatic cancer cell growth, while class II HDACs by themselves play minimal roles on this aspect. However, it is possible that simultaneous targeting of class I and class II HDACs may result in synergistic growth arrest of pancreatic cancer cells.

### Synergistic Antitumor Interactions between Class I- and Class II-Selective HDACIs in Pancreatic Cancer Cells

**Figure 3:**
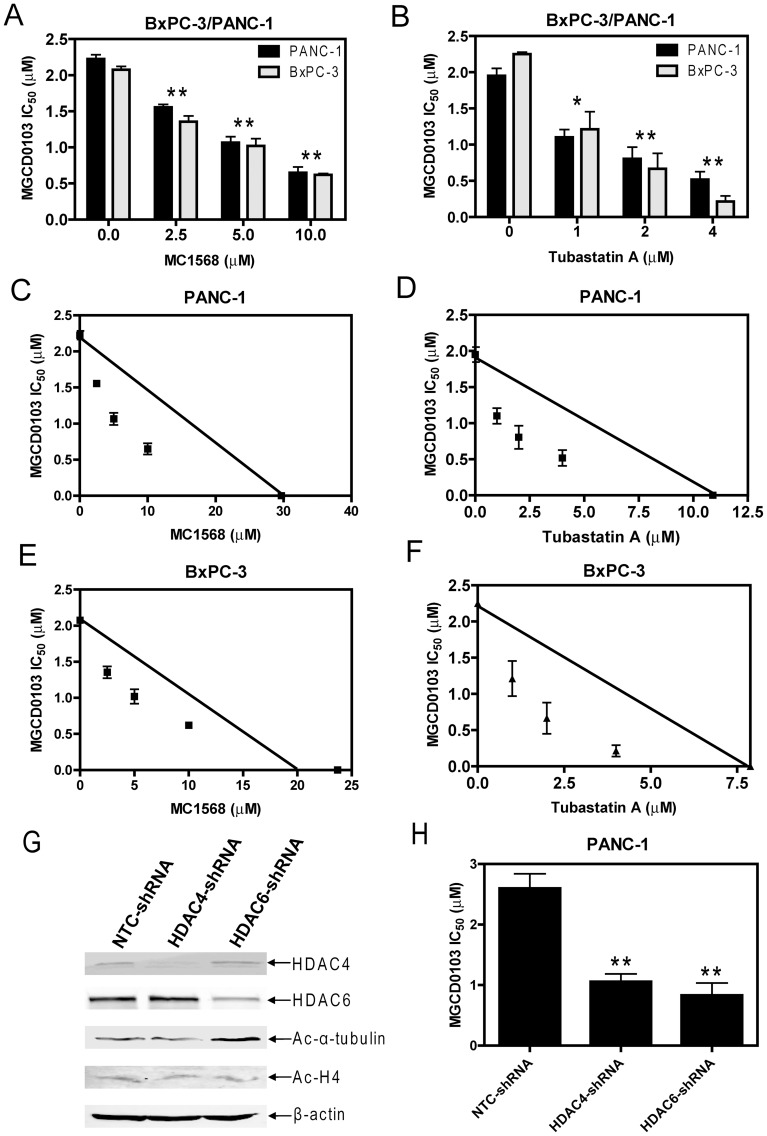
Synergistic interactions in inducing growth arrest between MGCD0103 and MC1568 or Tubastatin A in BxPC-3 or PANC-1 cells. **Panels A and B:** MGCD0103 IC_50_s of BxPC-3 or PANC-1 cells were determined in the absence or presence of MC1568 (panel A) or Tubastatin A (panel B) treated simultaneously. * indicates p<0.05, while ** indicates p<0.005. **Panels C–F:** Standard isobologram analysis of inhibition of PANC-1 (panels C&D) or BxPC-3 (panels E&F) cell growth by MGCD0103 and MC1568 (panels C&E) or Tubastatin A (panels D&F). The IC_50_ values of each drug are plotted on the axes; the solid line represents the additive effect, while the points represent the concentrations of each drug resulting in 50% inhibition of growth. Points falling below the line indicate synergism between drug combinations whereas those above the line indicate antagonism. **Panels G and H:** shRNA stable clones for a negative control (NTC), HDAC4, and HDAC6 were generated in PANC-1 cells. Expression levels of HDAC4, HDAC6, ac-H4 and ac-alpha-tubulin were determined by western blots (Panel G). Sensitivity to MGCD0103 of these shRNA stable clones was determined by MTT assays. ** indicates p<0.005.

**Figure 4:**
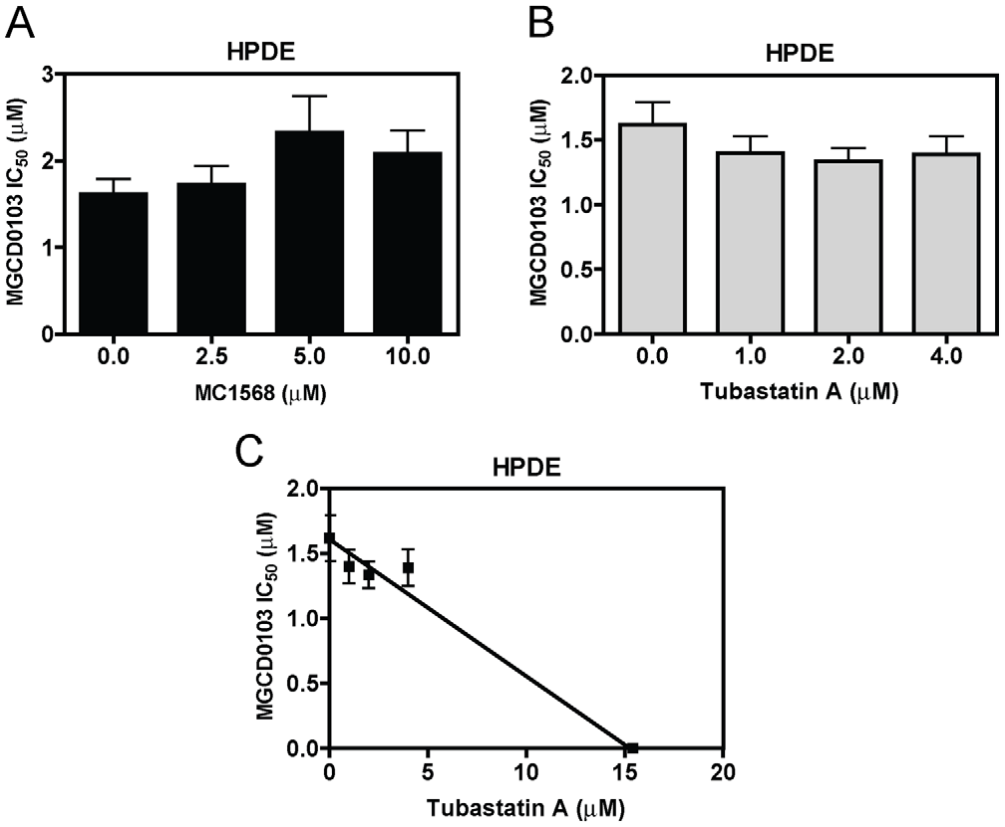
Interactions in inducing growth arrest between MGCD0103 and MC1568 or Tubastatin A in the normal HPDE cells. **Panels A and B:** MGCD0103 IC_50_s of HPDE cells were determined in the absence or presence of MC1568 (panel A) or Tubastatin A (panel B) treated simultaneously. **Panel C:** Standard isobologram analysis of inhibition of the HPDE cell growth by MGCD0103 and Tubastatin A. The IC_50_ values of each drug are plotted on the axes; the solid line represents the additive effect, while the points represent the concentrations of each drug resulting in 50% inhibition of growth. Points falling below the line indicate synergism between drug combinations whereas those above the line indicate antagonism.

To test this possibility, PANC-1 cells were treated with variable concentrations of MGCD0103, MC1568, or Tubastatin A, either alone or in combination for 96 h. Inhibition of cell growth by these treatments was measured by MTT assays. When administered simultaneously, MC1568 or Tubastatin A significantly enhanced MGCD0103-induced growth arrest (as reflected in the decreased IC_50_s) of the cells (Figures 3A&B). The combined effects of MGCD0103 with MC1568 or Tubastatin A on cell growth arrest were clearly synergistic, as determined by standard isobologram analyses (Figures 3C&D) and by calculating CI values with the CompuSyn software. A CI<1, indicative of synergism, was calculated for each of the drug combinations (data not shown). Almost identical results were obtained with BxPC-3 cells (Figures 3A, B, E, and F). To provide direct evidence that targeting class II HDACs can enhance the antitumor activity of MGCD0103 in pancreatic cancer cells, shRNA knockdown stable clones for HDAC4 (designated HDAC4-shRNA cells), HDAC6 (designated HDAC6-shRNA cells), and a negative control (designated NTC-shRNA cells) were generated in PANC-1 cells (Figure 3G). Interestingly, the HDAC4-shRNA and HDAC6-shRNA cells showed significantly increased sensitivities to MGCD0103 compared to the NTC-shRNA cells (MGCD0103 IC_50_s were 2.60, 1.06, and 0.83 µM for NTC-, HDAC4-, and HDAC6-shRNA cells, respectively; p<0.005) (Figure 3H). In great contrast, combined treatment of the HPDE cells with MC1568 and MGCD0103 resulted in slightly decreased MGCD0103 sensitivity (Figure 4A). Standard isobologram and CompuSyn analysis could not be performed due to the lack of response of the HPDE cells to MC1568. Although cotreatment of the HPDE cells with Tubastatin A and MGCD0103 also resulted in slightly increased sensitivity to MGCD0103 (Figure 4B), the interaction between the two agents was at the best additive when determined by standard isobologram analysis (Figure 4C).

**Figure 5:**
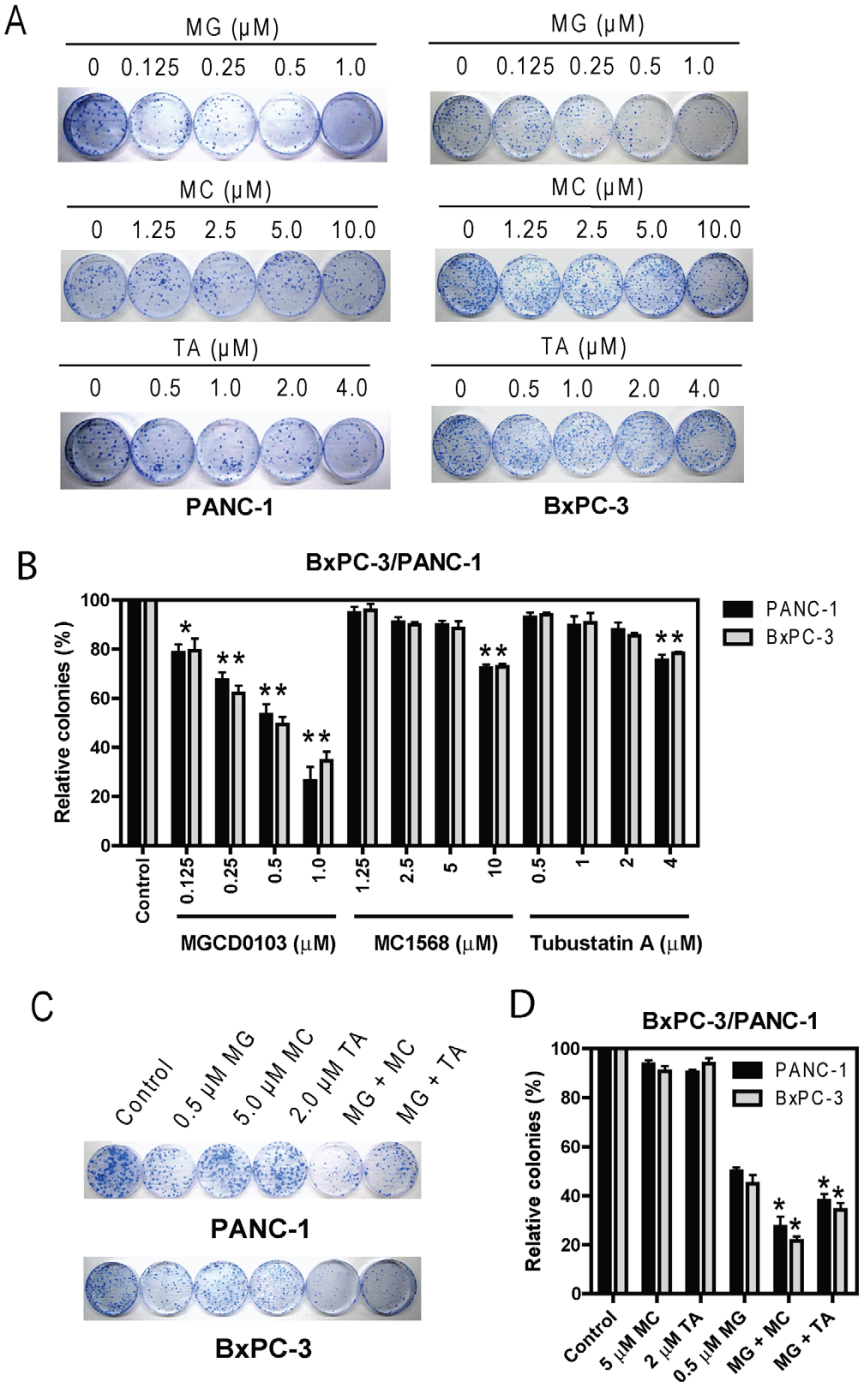
Synergistic interactions in inducing cell death between MGCD0103 and MC1568 or Tubastatin A in BxPC-3 or PANC-1 cells. Three hundred PANC-1 cells or five hundred BxPC-3 cells were plated in 100 mm dishes 1 day prior to the treatments with variable concentrations of MGCD0103, MC1568, or Tubastatin A, alone (**panels A and B**) or in combination (**panels C and D**) for 96 h. The drugs were then washed out, and the cells were cultured in drug-free complete medium for up to 3 weeks. Colonies were visualized by coomassie blue staining and counted. Results are presented as mean percentages ± standard errors relative to untreated control cells from three independent experiments. * indicates p<0.05, while ** indicates p<0.005.

Efforts were then undertaken to determine if class I- and class II-selective HDACIs synergize in causing pancreatic cancer cell death by colony formation assays. Treatments of PANC-1 or BxPC-3 cells with variable concentrations of MGCD0103 for 96 h resulted in dose-dependent induction of cell death, as reflected by the decreased numbers of colonies compared to untreated control cells (Figures 5A&B). This was in great contrast to the treatments with MC1568 or Tubastatin A which produced very limited effects on cell death, especially at lower concentrations (Figures 5A&B). Interestingly, when administered simultaneously, MC1568 or Tubastatin A significantly enhanced MGCD0103-induced cell death, as reflected by the further decreased numbers of colonies compared to that from MGCD0103 treatment alone (Figures 5C&D). These combined effects of MGCD0103 and MC1568 or Tubastatin A on the death of PANC-1 cells were synergistic when determined by using the CompuSyn software (CI = 0.46 and 0.77, respectively). Essentially the same results were obtained with BxPC-3 cells (CI = 0.30 and 0.54, respectively).

Taken together, our results suggest that class I HDACs play pivotal roles in pancreatic cancer cell growth and survival. Although class II HDACs by themselves play very limited roles, they cooperate with class I HDACs to enhance class I HDACs-mediated pancreatic cancer cell growth and survival.

### Effects of Class I- and Class II-Selective HDACIs on Apoptosis and Cell Cycle Progression in Pancreatic Cancer Cells

**Figure 6:**
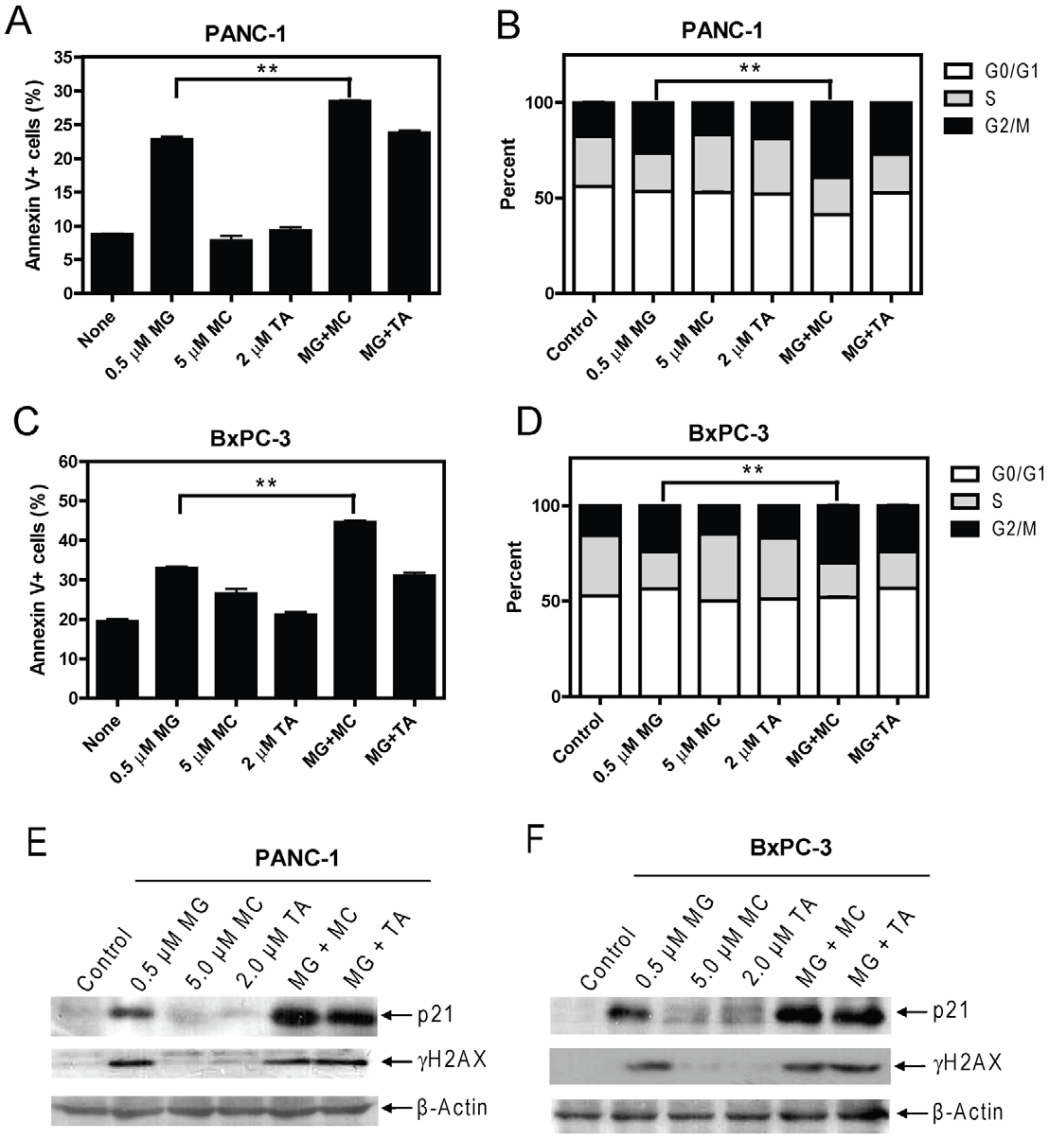
Panels A–D: Effects of class I- and class II-selective HDACIs on apoptosis and cell cycle progression in BxPC-3 and PANC-1 cells. PANC-1 (**panels A and B**) or BxPC-3 (**panels C and D**) cells were treated with MGCD0103, MC1568, or Tubastatin A, alone or combined for 96 h. The cells were harvested and subjected to flow cytometry analysis to measure both baseline and HDACI-induced apoptosis (**panels A and C**) and cell cycle distribution (**panels B and D**). The experiments were repeated three times, and results are presented as means ± standard errors of triplicates from one representative experiment. ** indicates p<0.005. **Panels E and F: Effects of class I- and class II-selective HDACIs on p21 expression and DNA DSBs in BxPC-3 and PANC-1 cells.** PANC-1 (**panel E**) or BxPC-3 (**panel F**) cells were treated with MGCD0103, MC1568, or Tubastatin A, alone or combined for 96 h. The cells were harvested and soluble proteins extracted and subjected to Western blotting to measure p21 and γH2AX levels. β-actin was used as the loading control.

To begin to determine the mechanisms by which MGCD0103 and MC1568 or Tubastatin A synergize in causing pancreatic cancer cell growth arrest and death, we next examined the effects of the three HDACIs on apoptosis and cell cycle distribution in PANC-1 and BxPC-3 cells. Treatments of PANC-1 or BxPC-3 cells with MGCD0103 (0.5 µM) resulted in induction of apoptosis and cell cycle arrest in G2/M phase. In contrast, treatments with MC1568 (5 µM) or Tubastatin A (2 µM) had no obvious effect on either apoptosis or cell cycle progression in these cells (Figures 6A–D). When combined simultaneously, MC1568 but not Tubastatin A, significantly enhanced MGCD0103-induced apoptosis and G2/M arrest in PANC-1 or BxPC-3 cells (Figures 6A–D).

These results suggest that inducing apoptosis and cell cycle arrest in G2/M phase may represent major mechanisms responsible for the cell death and growth arrest induced by MGCD0103, or MGCD0103 plus MC1568. Our results also suggest that class I HDACs play critical roles in pancreatic cancer cell apoptosis and G2 to M phase progression and these effects can be enhanced by class IIa HDACs, but not by HDAC6.

### Effects of Class I- and Class II-Selective HDACIs on DNA Double-Strand Breaks and p21 Expression in Pancreatic Cancer Cells

Recent studies demonstrated that inhibition of HDACs in cancer cells induces DNA damage, such as DNA double-strand breaks (DSBs), which can lead to activation of cell cycle checkpoints and subsequent apoptosis if the damaged DNA could not be repaired [42]–[45]. Further, HDACI-induced proliferation arrest is tightly linked to the induction of p21 [45]. Thus, HDACs may promote pancreatic cancer cell growth and survival through regulating p21 expression and DNA DSB repair. Interestingly, treatments of PANC-1 or BxPC-3 cells with MGCD0103 resulted in substantial induction of p21 and DNA DSBs, reflected in the induction of γH2AX, a biomarker of DSBs [46]. In contrast, treatments with MC1568 or Tubastatin A showed no effects on DNA DSBs or induction of p21. When combined simultaneously, both MC1568 and Tubastatin A cooperatively (if not synergistically) enhanced MGCD0103-induced expression of p21 (Figures 6E&F). However, these combinations did not show further effects on the levels of γH2AX compared to MGCD0103 treatment alone (Figures 6E&F). These results suggest that class II HDACs are required for maximal suppression of p21 expression predominantly mediated by class I HDACs, however, they are dispensable for class I HDACs-mediated repair of DNA DSBs.

## Discussion

HDACIs represent a promising new class of anticancer agents [19], [20], [45], [47]. Besides Vorinostat and Romidepsin which have been approved by the US Food and Drug Administration for treating cutaneous T-cell lymphoma, at least 11 other HDACIs are currently under clinical evaluation for treating both solid tumors and hematologic malignancies [19]. Although HDACIs have shown promising antitumor activities in preclinical models of pancreatic cancer [16], [21]–[24], it remains unclear which HDACs are the relevant therapeutic targets. The answer of this important question would be a prerequisite to the selection of the optimal HDACI for treating this extremely aggressive disease.

The aim of this study was to address the above question. We first determined the expression profiles of classes I and II HDACs in seven pancreatic cancer cell lines and normal HPDE cells. SIRTs 1–7 (class III HDACs) were excluded since traditional HDACIs don't inhibit this class of HDACs. Western blotting revealed that the majority of classes I and II HDACs (except for HDAC5) were readily detected in the pancreatic cancer cell lines, rendering them the potential to be involved in pancreatic cancer cell growth and/or survival. When compared to the normal HPDE cells, the levels of class I and class IIa HDACs in the majority of the pancreatic cancer cell lines were higher. These results suggest that targeting class I and class IIa HDACs by HDACIs for treating pancreatic cancer may have some level of tumor selectivity. However, this may not apply to class IIb HDACs since their levels in the HPDE cells were comparable to that in the pancreatic cancer cells.

We then used 3 different HDACIs with differential substrate specificities, MGCD0103, MC1568, and Tubastatin A, to explore the roles of class I and class II HDACs in pancreatic cancer cell growth and survival. Our MTT and colony formation assays suggested that class I HDACs play critical roles in promoting pancreatic cancer cell growth and survival. This is consistent with previous reports which highlight the importance of class I HDACs to cancer cell proliferation and survival which contrasts with class IIa HDACs 4 and 7 [25], [48], [49]. Surprisingly, the normal HPDE cells responded to MGCD0103 as well as the pancreatic cancer cell lines suggesting that the adverse effects of HDACIs observed clinically may be due to inhibition of class I HDACs. Although our results showed that selective targeting of class II HDACs resulted in minimal growth arrest and cell death, simultaneous targeting of both class I and class II HDACs with class I- and class II-selective HDACIs resulted in synergistic effects on both aspects in pancreatic cancer cells. In great contrast, these synergisms were not observed in the normal HPDE cells indicating that these drug combinations may not result in greater toxicity compared to that of MGCD0103 alone. Further, shRNA knockdown of HDAC4 and HDAC6 provided direct evidence that targeting class II HDACs can enhance the sensitivity of the class I-selective HDACI, MGCD0103, in PANC-1 cells. Thus, our results support the notion that both classes I and II HDACs are potential therapeutic targets for treating pancreatic cancer. This novel finding is crucial for selecting the optimal HDACI for treating the disease.

We next began to determine the mechanisms underlying the synergistic antitumor interactions between class I- and class II-selective HDACIs in pancreatic cancer cells. Flow cytometry analyses revealed that induction of apoptosis and cell cycle arrest in G2/M phase may be responsible for the antitumor effects of MGCD0103. This was accompanied by induction of DNA DSBs (reflected in the induction of γH2AX) and p21 expression in the cells. Consistent with our results from MTT and colony formation assays, treatments with MC1568 or Tubastatin A did not result in obvious effects on apoptosis, cell cycle progression, or induction of DNA DSBs and p21 expression in both BxPC-3 and PANC-1 cells. These results are consistent with previous studies suggesting that HDACs 4 and 7 are not important for cancer cell proliferation and survival, however, differ from a recent study which showed DNA damage induction in cancer cells by selective targeting of HDAC6 [25], [50]. This difference could be attributed to the different cancer cell lines used in the studies. When combined simultaneously, MC1568 (5 µM, the minimum dose to induce maximum acetylation of histone H4 in PANC-1 cells) significantly enhanced MGCD0103-induced apoptosis and G2/M arrest in both cell lines, accompanied by cooperative induction of p21, but not γH2AX. These results suggest that class I HDACs play primary roles in modulating apoptosis, cell cycle progression from G2 to M, DNA DSB repair, and p21 expression in pancreatic cancer cells. Although class IIa HDACs by themselves seem not to play a role on these aspects, they cooperate with class I HDACs to promote pancreatic cancer cell growth and survival potentially mediated by mechanisms involving apoptosis and cell cycle progression from G2 to M, independent of the repair of DNA DSBs. Although Tubastatin A (at 2 µM, the minimum dose to induce maximum acetylation of alpha-tubulin) also cooperated with MGCD0103 in inducing p21, but not γH2AX, it had no effects on MGCD0103-induced apoptosis and cell cycle arrest in G2/M in the cells. Thus, other mechanisms must exist responsible for the enhancing effects of HDAC6 on class I HDACs-mediated pancreatic cancer cell growth and survival.

Together, we report for the first time that both class I- and class II-selective HDACIs synergize in inducing growth arrest and death of pancreatic cancer cells, but not in normal HPDE cells. However, the molecular mechanisms underlying the synergistic antitumor interactions between class I and class II HDACIs are not entirely clear, which warrant further investigation. Further, our *in vitro* findings need follow up studies in *in vivo* models. Nonetheless, our data suggest that both classes I and II HDACs are potential therapeutic targets for treating the disease. Accordingly, treating pancreatic cancer with a true pan-HDACI (which targets both classes I, IIa and IIb HDACs) or with combined class I and class II HDACIs may be more beneficial than the use of class- or isoform-selective HDACIs.

## References

[1] Siegel R, Ward E, Brawley O, Jemal A (2011) Cancer statistics, 2011: the impact of eliminating socioeconomic and racial disparities on premature cancer deaths. CA Cancer J Clin 61: 212–236. 21685461 10.3322/caac.20121

[2] Giovannetti E, Mey V, Nannizzi S, Pasqualetti G, Del Tacca M, et al (2006) Pharmacogenetics of anticancer drug sensitivity in pancreatic cancer. Mol Cancer Ther 5: 1387–1395. 16818496 10.1158/1535-7163.MCT-06-0004

[3] Strimpakos A, Saif MW, Syrigos KN (2008) Pancreatic cancer: from molecular pathogenesis to targeted therapy. Cancer Metastasis Rev 27: 495–522. 18427734 10.1007/s10555-008-9134-y

[4] Burris HA 3rd, Moore MJ, Andersen J, Green MR, Rothenberg ML, et al (1997) Improvements in survival and clinical benefit with gemcitabine as first-line therapy for patients with advanced pancreas cancer: a randomized trial. J Clin Oncol 15: 2403–2413. 9196156 10.1200/JCO.1997.15.6.2403

[5] Kullmann F, Hollerbach S, Dollinger MM, Harder J, Fuchs M, et al (2009) Cetuximab plus gemcitabine/oxaliplatin (GEMOXCET) in first-line metastatic pancreatic cancer: a multicentre phase II study. Br J Cancer 100: 1032–1036. 19293797 10.1038/sj.bjc.6604983 PMC2670003

[6] Li J, Saif MW (2009) Advancements in the management of pancreatic cancer. JOP 10: 109–117. 19287102

[7] Carew JS, Giles FJ, Nawrocki ST (2008) Histone deacetylase inhibitors: mechanisms of cell death and promise in combination cancer therapy. Cancer Lett 269: 7–17. 18462867 10.1016/j.canlet.2008.03.037

[8] Witt O, Deubzer HE, Milde T, Oehme I (2009) HDAC family: What are the cancer relevant targets? Cancer Lett 277: 8–21. 18824292 10.1016/j.canlet.2008.08.016

[9] de Ruijter AJ, van Gennip AH, Caron HN, Kemp S, van Kuilenburg AB (2003) Histone deacetylases (HDACs): characterization of the classical HDAC family. Biochem J 370: 737–749. 12429021 10.1042/BJ20021321 PMC1223209

[10] Gregoretti IV, Lee YM, Goodson HV (2004) Molecular evolution of the histone deacetylase family: functional implications of phylogenetic analysis. J Mol Biol 338: 17–31. 15050820 10.1016/j.jmb.2004.02.006

[11] Verdin E, Dequiedt F, Kasler HG (2003) Class II histone deacetylases: versatile regulators. Trends Genet 19: 286–293. 12711221 10.1016/S0168-9525(03)00073-8

[12] Guardiola AR, Yao TP (2002) Molecular cloning and characterization of a novel histone deacetylase HDAC10. J Biol Chem 277: 3350–3356. 11726666 10.1074/jbc.M109861200

[13] Blander G, Guarente L (2004) The Sir2 family of protein deacetylases. Annu Rev Biochem 73: 417–435. 15189148 10.1146/annurev.biochem.73.011303.073651

[14] Ledent V, Vervoort M (2006) Comparative genomics of the class 4 histone deacetylase family indicates a complex evolutionary history. BMC Biol 4: 24. 16884538 10.1186/1741-7007-4-24 PMC1555614

[15] Feinberg AP, Ohlsson R, Henikoff S (2006) The epigenetic progenitor origin of human cancer. Nat Rev Genet 7: 21–33. 16369569 10.1038/nrg1748

[16] Sung V, Richard N, Brady H, Maier A, Kelter G, et al (2011) Histone deacetylase inhibitor MGCD0103 synergizes with gemcitabine in human pancreatic cells. Cancer Sci 102: 1201–1207. 21375679 10.1111/j.1349-7006.2011.01921.x

[17] Sasaki H, Moriyama S, Nakashima Y, Kobayashi Y, Kiriyama M, et al (2004) Histone deacetylase 1 mRNA expression in lung cancer. Lung Cancer 46: 171–178. 15474665 10.1016/j.lungcan.2004.03.021

[18] Miyake K, Yoshizumi T, Imura S, Sugimoto K, Batmunkh E, et al (2008) Expression of hypoxia-inducible factor-1alpha, histone deacetylase 1, and metastasis-associated protein 1 in pancreatic carcinoma: correlation with poor prognosis with possible regulation. Pancreas 36: e1–9. 10.1097/MPA.0b013e31815f2c2a 18362831

[19] Marks PA (2010) The clinical development of histone deacetylase inhibitors as targeted anticancer drugs. Expert Opin Investig Drugs 19: 1049–1066. 10.1517/13543784.2010.510514 PMC4077324 20687783

[20] Bolden JE, Peart MJ, Johnstone RW (2006) Anticancer activities of histone deacetylase inhibitors. Nat Rev Drug Discov 5: 769–784. 16955068 10.1038/nrd2133

[21] Chun SG, Zhou W, Yee NS (2009) Combined targeting of histone deacetylases and hedgehog signaling enhances cytoxicity in pancreatic cancer. Cancer Biol Ther 8: 1328–1339. 19421011 10.4161/cbt.8.14.8633

[22] Kumagai T, Wakimoto N, Yin D, Gery S, Kawamata N, et al (2007) Histone deacetylase inhibitor, suberoylanilide hydroxamic acid (Vorinostat, SAHA) profoundly inhibits the growth of human pancreatic cancer cells. Int J Cancer 121: 656–665. 17417771 10.1002/ijc.22558

[23] Arnold NB, Arkus N, Gunn J, Korc M (2007) The histone deacetylase inhibitor suberoylanilide hydroxamic acid induces growth inhibition and enhances gemcitabine-induced cell death in pancreatic cancer. Clin Cancer Res 13: 18–26. 17200334 10.1158/1078-0432.CCR-06-0914

[24] Kauh J, Fan S, Xia M, Yue P, Yang L, et al (2010) c-FLIP degradation mediates sensitization of pancreatic cancer cells to TRAIL-induced apoptosis by the histone deacetylase inhibitor LBH589. PLoS One 5: e10376. 20442774 10.1371/journal.pone.0010376 PMC2860986

[25] Glaser KB, Li J, Staver MJ, Wei RQ, Albert DH, et al (2003) Role of class I and class II histone deacetylases in carcinoma cells using siRNA. Biochem Biophys Res Commun 310: 529–536. 14521942 10.1016/j.bbrc.2003.09.043

[26] Lagger G, O'Carroll D, Rembold M, Khier H, Tischler J, et al (2002) Essential function of histone deacetylase 1 in proliferation control and CDK inhibitor repression. EMBO J 21: 2672–2681. 12032080 10.1093/emboj/21.11.2672 PMC126040

[27] Bali P, Pranpat M, Bradner J, Balasis M, Fiskus W, et al (2005) Inhibition of histone deacetylase 6 acetylates and disrupts the chaperone function of heat shock protein 90: a novel basis for antileukemia activity of histone deacetylase inhibitors. J Biol Chem 280: 26729–26734. 15937340 10.1074/jbc.C500186200

[28] Bradner JE, West N, Grachan ML, Greenberg EF, Haggarty SJ, et al (2010) Chemical phylogenetics of histone deacetylases. Nat Chem Biol 6: 238–243. 20139990 10.1038/nchembio.313 PMC2822059

[29] Khan N, Jeffers M, Kumar S, Hackett C, Boldog F, et al (2008) Determination of the class and isoform selectivity of small-molecule histone deacetylase inhibitors. Biochem J 409: 581–589. 17868033 10.1042/BJ20070779

[30] Duong V, Bret C, Altucci L, Mai A, Duraffourd C, et al (2008) Specific activity of class II histone deacetylases in human breast cancer cells. Mol Cancer Res 6: 1908–1919. 19074835 10.1158/1541-7786.MCR-08-0299 PMC2810315

[31] Mai A, Massa S, Pezzi R, Rotili D, Loidl P, et al (2003) Discovery of (aryloxopropenyl)pyrrolyl hydroxyamides as selective inhibitors of class IIa histone deacetylase homologue HD1-A. J Med Chem 46: 4826–4829. 14584932 10.1021/jm034167p

[32] Mai A, Massa S, Pezzi R, Simeoni S, Rotili D, et al (2005) Class II (IIa)-selective histone deacetylase inhibitors. 1. Synthesis and biological evaluation of novel (aryloxopropenyl)pyrrolyl hydroxyamides. J Med Chem 48: 3344–3353. 15857140 10.1021/jm049002a

[33] Butler KV, Kalin J, Brochier C, Vistoli G, Langley B, et al (2010) Rational design and simple chemistry yield a superior, neuroprotective HDAC6 inhibitor, tubastatin A. J Am Chem Soc. 132: 10842–10846. 10.1021/ja102758v PMC2916045 20614936

[34] Deer EL, Gonzalez-Hernandez J, Coursen JD, Shea JE, Ngatia J, et al (2010) Phenotype and genotype of pancreatic cancer cell lines. Pancreas 39: 425–435. 20418756 10.1097/MPA.0b013e3181c15963 PMC2860631

[35] Xie C, Edwards H, Xu X, Zhou H, Buck SA, et al (2010) Mechanisms of synergistic antileukemic interactions between valproic acid and cytarabine in pediatric acute myeloid leukemia. Clin Cancer Res 16: 5499–5510. 20889917 10.1158/1078-0432.CCR-10-1707 PMC3018695

[36] Xu X, Xie C, Edwards H, Zhou H, Buck SA, et al (2011) Inhibition of histone deacetylases 1 and 6 enhances cytarabine-induced apoptosis in pediatric acute myeloid leukemia cells. PLoS One 6: e17138. 21359182 10.1371/journal.pone.0017138 PMC3040224

[37] Chou TC (2006) Theoretical basis, experimental design, and computerized simulation of synergism and antagonism in drug combination studies. Pharmacol Rev 58: 621–681. 16968952 10.1124/pr.58.3.10

[38] Tallarida RJ (2001) Drug synergism: its detection and applications. J Pharmacol Exp Ther 298: 865–872. 11504778

[39] Edwards H, Xie C, LaFiura KM, Dombkowski AA, Buck SA, et al (2009) RUNX1 regulates phosphoinositide 3-kinase/AKT pathway: role in chemotherapy sensitivity in acute megakaryocytic leukemia. Blood 114: 2744–2752. 19638627 10.1182/blood-2008-09-179812 PMC2756129

[40] Ge Y, Stout ML, Tatman DA, Jensen TL, Buck S, et al (2005) GATA1, cytidine deaminase, and the high cure rate of Down syndrome children with acute megakaryocytic leukemia. J Natl Cancer Inst 97: 226–231. 15687366 10.1093/jnci/dji026

[41] Xie C, Edwards H, Lograsso SB, Buck SA, Matherly LH, et al (2012) Valproic acid synergistically enhances the cytotoxicity of clofarabine in pediatric acute myeloid leukemia cells. Pediatr Blood Cancer 59: 1245–1251. 22488775 10.1002/pbc.24152 PMC3396758

[42] Conti C, Leo E, Eichler GS, Sordet O, Martin MM, et al (2010) Inhibition of histone deacetylase in cancer cells slows down replication forks, activates dormant origins, and induces DNA damage. Cancer Res 70: 4470–4480. 20460513 10.1158/0008-5472.CAN-09-3028 PMC2880188

[43] Gaymes TJ, Padua RA, Pla M, Orr S, Omidvar N, et al (2006) Histone deacetylase inhibitors (HDI) cause DNA damage in leukemia cells: a mechanism for leukemia-specific HDI-dependent apoptosis? Mol Cancer Res 4: 563–573. 16877702 10.1158/1541-7786.MCR-06-0111

[44] Dai Y, Grant S (2010) New insights into checkpoint kinase 1 in the DNA damage response signaling network. Clin Cancer Res 16: 376–383. 20068082 10.1158/1078-0432.CCR-09-1029 PMC2939735

[45] Minucci S, Pelicci PG (2006) Histone deacetylase inhibitors and the promise of epigenetic (and more) treatments for cancer. Nat Rev Cancer 6: 38–51. 16397526 10.1038/nrc1779

[46] Redon CE, Nakamura AJ, Zhang YW, Ji JJ, Bonner WM, et al (2010) Histone gammaH2AX and poly(ADP-ribose) as clinical pharmacodynamic biomarkers. Clin Cancer Res 16: 4532–4542. 20823146 10.1158/1078-0432.CCR-10-0523 PMC2940983

[47] Prince HM, Bishton MJ, Harrison SJ (2009) Clinical studies of histone deacetylase inhibitors. Clin Cancer Res 15: 3958–3969. 19509172 10.1158/1078-0432.CCR-08-2785

[48] Huang BH, Laban M, Leung CH, Lee L, Lee CK, et al (2005) Inhibition of histone deacetylase 2 increases apoptosis and p21Cip1/WAF1 expression, independent of histone deacetylase 1. Cell Death Differ 12: 395–404. 15665816 10.1038/sj.cdd.4401567

[49] Karagiannis TC, El-Osta A (2007) Will broad-spectrum histone deacetylase inhibitors be superseded by more specific compounds? Leukemia 21: 61–65. 17109024 10.1038/sj.leu.2404464

[50] Namdar M, Perez G, Ngo L, Marks PA (2010) Selective inhibition of histone deacetylase 6 (HDAC6) induces DNA damage and sensitizes transformed cells to anticancer agents. Proc Natl Acad Sci U S A 107: 20003–20008. 21037108 10.1073/pnas.1013754107 PMC2993347

